# Novel reassortant clade 2.3.4.4 avian influenza A (H5N8) virus in a grey heron in South Korea in 2017

**DOI:** 10.1007/s00705-017-3547-2

**Published:** 2017-09-12

**Authors:** Chanjin Woo, Jung-Hoon Kwon, Dong-Hun Lee, Youngsik Kim, Kwanghee Lee, Seong-Deok Jo, Ki dong Son, Jae-Ku Oem, Seung-Jun Wang, Yongkwan Kim, Jeonghwa Shin, Chang-Seon Song, Weonhwa Jheong, Jipseol Jeong

**Affiliations:** 1 Environmental Health Research Department, National Institute of Environmental Research, Hwangyeong-ro 42, Seo-gu, Incheon, Republic of Korea; 2 Avian Disease Laboratory, College of Veterinary Medicine, Konkuk University, Neungdong-ro 120, Gwangjin-gu, Seoul, Republic of Korea; 3 Southeast Poultry Research Laboratory, U.S. National Poultry Research Center, Agricultural Research Service, U.S. Department of Agriculture, Athens, GA USA

**Keywords:** Avian influenza virus, Wild bird, H5N8, Clade 2.3.4.4, Influenza

## Abstract

We report the identification of a novel reassortant clade 2.3.4.4 H5N8 virus from a dead grey heron in Korea in 2017. Outbreaks of clade 2.3.4.4 H5 HPAIVs have been reported worldwide, and they have evolved into multiple genotypes among wild birds. Phylogenetic analysis indicated that this virus likely originated from Qinghai Lake and Western Siberia and further evolved through reassortment with Eurasian LPAI during the 2016 fall migration of wild birds. Enhanced surveillance and comparative genetic analysis will help to monitor the further evolution and dissemination of clade 2.3.4.4 HPAIVs.

**Electronic supplementary material:**

The online version of this article (doi:10.1007/s00705-017-3547-2) contains supplementary material, which is available to authorized users.

Avian influenza virus (AIV) belongs to the genus *Influenzavirus A* of the family *Orthomyxoviridae*. Sixteen hemagglutinin (HA) subtypes (H1 to H16) and nine neuraminidase (NA) subtypes (N1 to N9) have been identified in avian species. To date, all identified highly pathogenic AIVs (HPAIVs) that cause high mortality in poultry have been reported to be of the H5 or H7 subtype. Since 1996, the A/goose/Guangdong/1/1996 (Gs/Gd) lineage of H5 HPAIV has spread to more than 60 countries and resulted in outbreaks with considerable economic losses to the poultry industry, infections in wild birds, and clinical, often fatal, cases in humans [1].

The Gs/Gd H5 HPAIVs have evolved into 10 genetically distinct HA clades (0–9) and have undergone reassortment with neuraminidase and internal genes from other viruses to generate novel reassortant viruses with NA gene subtypes N1, N2, N3, N5, N6, N8, and N9 [2–4]. The HA gene of clade 2.3.4.4 H5 HPAIVs has evolved into four distinct genetic groups, A-D [5]. In early 2014, outbreaks of groups A and B of H5N8 clade 2.3.4.4 were reported in South Korea, followed by outbreaks of group A H5N8 HPAI in Europe and North America in late 2014 [6, 7]. Aquatic migratory birds are suspected to play a key role in the global spread of group A H5N8 clade 2.3.4.4 from East Asia to Europe and North America [8]. In contrast, there have been less widespread reports of the group B viruses since their original detection in China in 2013 and South Korea in early 2014 [6].

In May 2016, novel reassortant group B clade 2.3.4.4 H5N8 viruses were detected in wild birds at Qinghai Lake in China and Lake Uvs-Nuur at the Russia-Mongolia border, and they were subsequently detected in various regions of Europe, Africa, and India in the fall of 2016 [9–12]. Meanwhile, novel reassortant group C clade 2.3.4.4 H5N6 viruses were identified in Korea and Japan in the fall of 2016, and they have since caused numerous outbreaks in domestic poultry and wild birds in Korea [13, 14]. In January 2017, during HPAI surveillance of wild birds in Korea, we isolated a group B H5N8 clade 2.3.4.4 virus from the carcass of a grey heron (*Ardea cinerea*). In this study, we sequenced this H5N8 virus, A/grey heron/Korea/W779/2017(H5N8) (W779), and conducted a comparative phylogenetic analysis to trace its origin and understand its genetic relationship to other group B H5N8 clade 2.3.4.4 viruses identified in Eurasia and Africa in 2016-2017.

The dead grey heron from which we isolated A/grey heron/Korea/W779/2017(H5N8) was found in Jeonju, South Korea (35°52’4.15”N, 127°6’28.08”E), on January 27, 2017. Tissue samples were collected from the carcass and homogenized with phosphate-buffered saline (PBS) to a 10% (wt/vol) final concentration. The swabs were collected into 1 ml of antibiotic-containing PBS (gentamicin, 250 μg/mL). After centrifugation, 100 μl of medium from each tissue and swab sample was inoculated into the allantoic cavity of a 10-day-old specific-pathogen-free (SPF) embryonated chicken egg. The allantoic fluid was harvested after 72 h of incubation at 37 °C. AIV was identified by a hemagglutination assay and an H5 subtype-specific reverse transcription polymerase chain reaction (RT-PCR) assay [15]. The isolate was identified as H5N8 AIV by subtyping RT-PCR [16]. The complete genomes were sequenced using influenza A universal primers as described previously [17]. Nucleotide sequences were deposited in the GenBank database (accession nos. MF155629-MF155636). For phylogenetic analysis, by reference sequences were selected based on sequence homology as determined by GISAID EpiFLU BLAST searches (http://platform.gisaid.org), including clade 2.3.4.4 H5 HPAIVs identified in 2016-2017 and related low-pathogenic strains [3]. For Bayesian phylogenetic analysis and estimation of the time to the most recent common ancestor (tMRCA), Bayesian analysis was performed for all eight gene segments using BEAST version 1.8.4. We employed a Hasegawa-Kishino-Yano (HKY) substitution model with four gamma categories and specified an uncorrelated lognormal relaxed clock and GMRF Bayesian skyride tree prior for each segment. The Markov chain Monte Carlo (MCMC) method was employed with 50 million chain lengths to draw inference under this model. BEAST output was analyzed with TRACER v1.4 (https://beast.bio.ed.ac.uk/tracer) with 10% burn-in. Maximum-clade-credibility (MCC) trees with median heights were generated for each dataset using TreeAnnotator version 1.8.4 and visualized using the program FigTree v1.4.2. Estimated tMRCA values were obtained from MCC trees with common ancestor heights.

We considered the W779 virus to be an HPAIV on the basis of the deduced amino acid sequence at the HA proteolytic cleavage site (PLREKRRKR/G). In the HA, the receptor-binding sites maintained Q226 and G228 (H3 numbering), which is suggestive of preferential binding to the sialic acid-2,3-NeuAcGal, as is typical for avian influenza viruses [18]. The established markers of NA inhibitor resistance (E119A, H274Y, and N294S, N2 numbering) and amantadine resistance (V27A and S31N, M2 protein) were not found [19, 20]. In addition, phenotypic markers related to increased potential for transmission and pathogenicity to mammals such as markers in PB2 (E158G, E627K, and K701N), PB1 (Y436H), and PA (T515A) were also not found [21, 22].

Phylogenetic analysis of the HA gene showed that the W779 virus clustered within group B of H5 clade 2.3.4.4 HPAIV (Fig. 1 and Supplemental Figure 1). However, the characterization of the complete genome revealed that this virus had likely undergone reassortment with Eurasian low-pathogenic AIVs (LPAIVs). Particularly, the PB2, PB1, HA, NA, M, and NS genes of the W779 virus clustered with group B H5N8 clade 2.3.4.4 HPAIV identified in Western Siberia (Uvs-Nuur Lake), China (Qinghai Lake), Europe, and India in 2016-2017 (Supplemental Figures 1 and 2). On the other hand, the PA gene of the W779 virus was phylogenetically distinct from those of H5N8 viruses from Qinghai Lake and Uvs-Nuur Lake but clustered with an Indian isolate (A/painted stork/India/10CA03/2016, India 10CA03), an Italian isolate (A/turkey/Italy/17VIR538-1/2017), and a Russian isolate (A/gadwall/Chany/97/2016, Chany 97). The NP gene was phylogenetically distinct from H5N8 viruses identified at Qinghai Lake, Uvs-Nuur Lake, India (India 10CA03), Russia (Chany 97), and Europe.

**Fig. 1:**
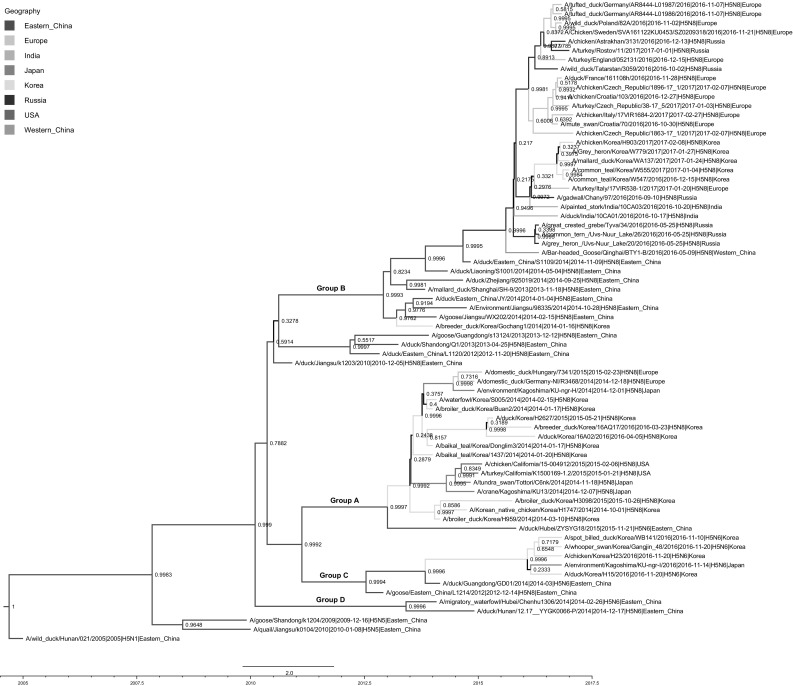
Temporally structured maximum-clade-credibility phylogenetic tree (years on the horizontal axis) of the hemagglutinin gene of HPAIV H5 clade 2.3.4.4 viruses. The Korean 2017 H5N8 isolate used in this study is colored in red. Branches are colored according to location. The posterior probabilities of Bayesian analysis in which the associated taxa clustered together are shown next to the branches

We estimated that ancestors of the novel H5N8 HPAIV identified in Korea emerged among wild birds during May-September 2016, based on the tMRCA for each gene segment of H5N8 HPAIV identified in Korea during December 2016-February 2017 (Table 1), which corresponds to timing of HPAIV detection in Qinghai Lake, Western Siberia, and Russia. Based on these data, we speculate that the W779 virus is a descendant of group B H5N8 viruses identified in waterfowl in Siberia (or nearby high latitudes) during May-September 2016 that acquired PA and NP segments from a gene pool of Eurasian LPAIV before detection in Korea in the winter of 2016-2017. All genes of A/grey heron/Korea/W779/2017(H5N8) were phylogenetically distinct from group A of clade 2.3.4.4 H5N8 viruses that circulated in South Korea in 2014-2016 and group C of clade 2.3.4.4 H5N6 viruses isolated in South Korea in 2016. Collectively, the phylogenetic analysis indicated that the W779 virus is a novel reassortant virus of group B clade 2.3.4.4 H5N8 that shares genetic ancestry with H5N8 HPAIV from Qinghai Lake and Western Siberia and likely evolved through reassortment with Eurasian LPAIVs.

**Table 1:** Time of the most recent common ancestor for each gene segment of H5N8 viruses isolated in Korea, 2016-2017

	Mean	95% HPD	Posterior probability
PB2	August 2016	June – October 2016	0.9992
PB1	August 2016	July – October 2016	0.9993
PA	July 2016	May – October 2016	0.9995
HA	September 2016	July – October 2016	0.9997
NP	May 2016	October 2015 – October 2016	0.9997
NA	May 2016	June – October 2016	0.9689
M	August 2016	June – October 2016	0.4849
NS	September 2016	June – October 2016	0.9995

Previous studies also suggested that group B clade 2.3.4.4 H5N8 viruses identified from stopover and wintering regions of wild birds (Europe, India, and Russia) in fall and winter 2016 were genetically close to H5N8 viruses identified from breeding regions in Western Siberia during spring 2016 [9–12]. The W779 virus had a different genome constellation from other reassortant H5N8 viruses identified in Europe, Russia and India during 2016-2017, suggesting that the W779 virus evolved by an independent reassortment event from other reassortant H5N8 viruses (Supplemental Figure 1).

It is believed that wild birds have played an important role in dissemination of Gs/Gd HPAIV, as seen in the spread of clade 2.2 H5N1 HPAIV from Qinghai Lake and circumpolar breeding areas to Europe and East Asia in 2005-2006 [23, 24] and clade 2.3.4.4 H5N8 HPAIV from Siberia to Europe, Asia, and North America in 2014 [8]. Based on previous reports, possible long-distance dissemination of virus via wild bird migration, and our phylogenetic analysis in this study, we suspected that migratory wild birds introduced the novel reassortant H5N8 HPAIVs into Korea. Since 2014, outbreaks of clade 2.3.4.4 H5 HPAIV have been reported in various geographic regions, and they have evolved into multiple genotypes [5]. Enhanced surveillance and comparative genetic analysis will help to monitor the further evolution and dissemination of clade 2.3.4.4 HPAIVs.

## Electronic supplementary material

Below is the link to the electronic supplementary material.

**Supplemental Figure 1** Temporally structured maximum-clade-credibility phylogenetic tree (years on the horizontal axis) of the PB2, PB1, PA, HA, NP, NA, M, and NS genes of HPAIV H5 clade 2.3.4.4 viruses. The Korean 2017 H5N8 isolate used in this study is colored in red. The posterior probabilities of Bayesian analysis in which the associated taxa clustered together are shown next to the branches. (PDF 7 kb)

Supplementary material 2 (PDF 7 kb)

Supplementary material 3 (PDF 8 kb)

Supplementary material 4 (PDF 6 kb)

Supplementary material 5 (PDF 8 kb)

Supplementary material 6 (PDF 8 kb)

Supplementary material 7 (PDF 9 kb)
