# Supplementary figures and images for: Novel reassortant clade 2.3.4.4 avian influenza A (H5N8) virus in a grey heron in South Korea in 2017
Source: Arch Virol. 2017 Sep 12;162(12):3887–91. doi: 10.1007/s00705-017-3547-2 (PMC5671518; doi:10.1007/s00705-017-3547-2)

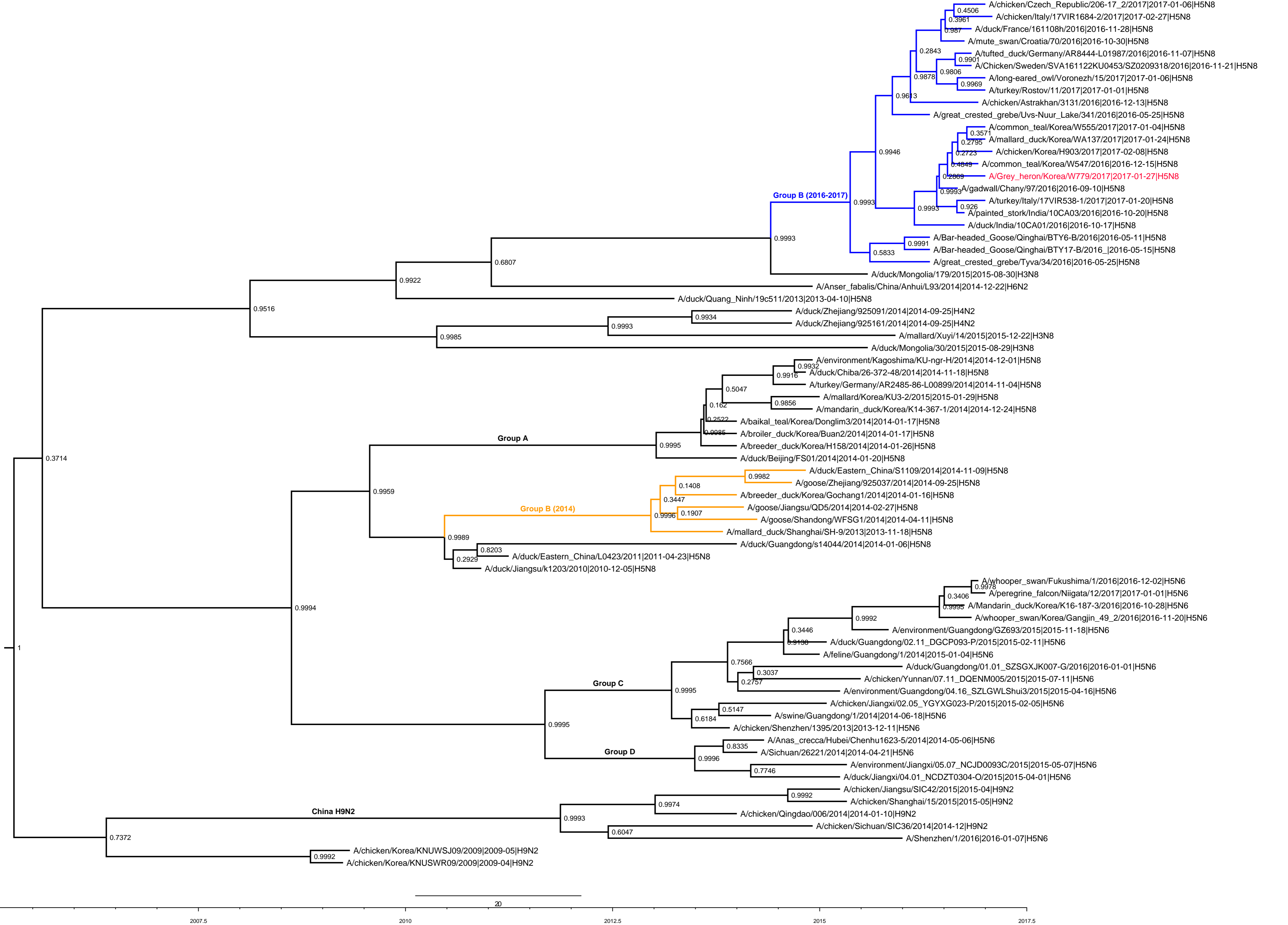

Supplement: Supplementary file 1 — Supplemental Figure 1 Temporally structured maximum-clade-credibility phylogenetic tree (years on the horizontal axis) of the PB2, PB1, PA, HA, NP, NA, M, and NS genes of HPAIV H5 clade 2.3.4.4 viruses. The Korean 2017 H5N8 isolate used in this study is colored in red. The posterior probabilities of Bayesian analysis in which the associated taxa clustered together are shown next to the branches. (PDF 7 kb) [file 705_2017_3547_MOESM1_ESM.pdf]

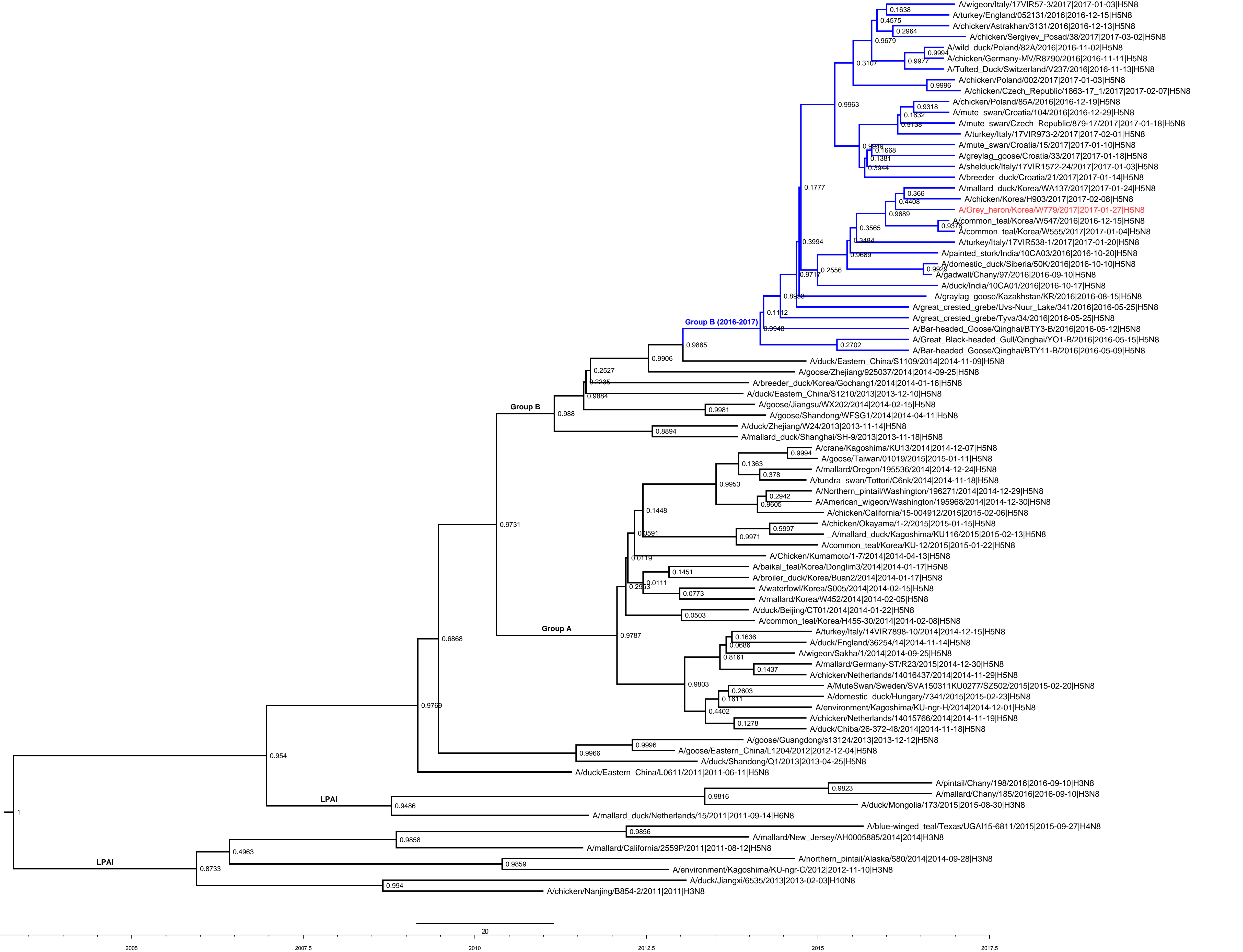

Supplement: Supplementary file 2 — Supplementary material 2 (PDF 7 kb) [file 705_2017_3547_MOESM2_ESM.pdf]

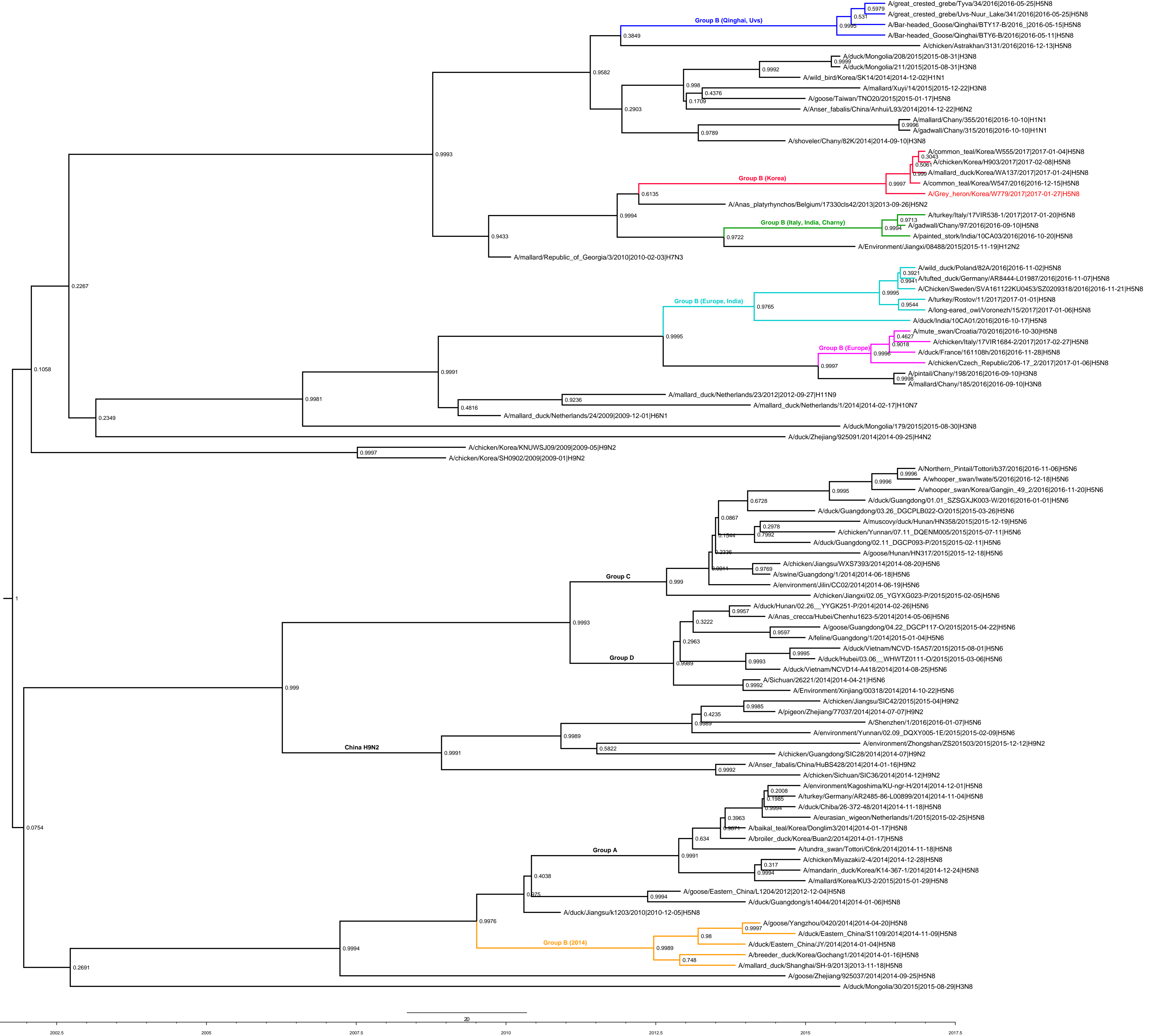

Supplement: Supplementary file 3 — Supplementary material 3 (PDF 8 kb) [file 705_2017_3547_MOESM3_ESM.pdf]

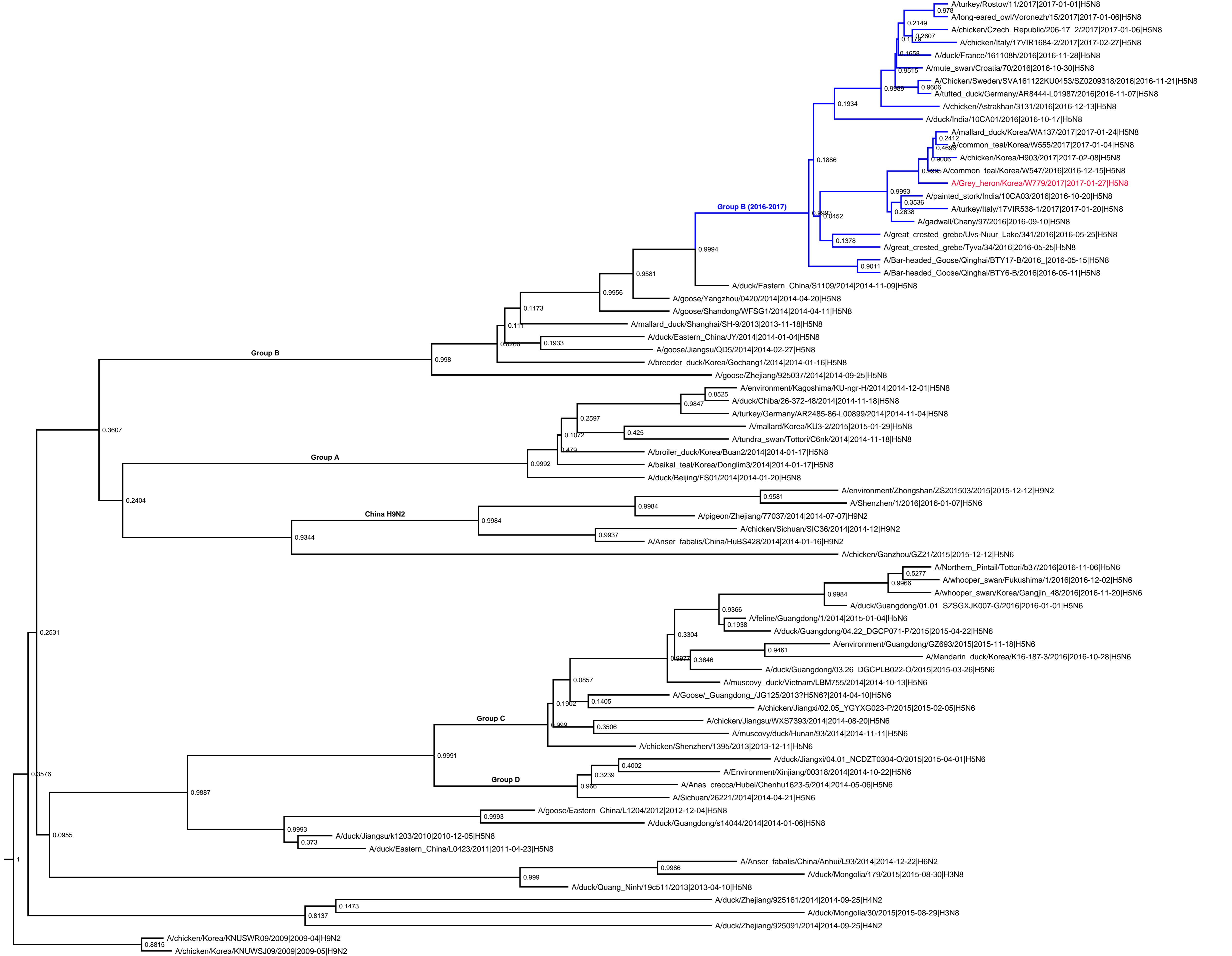

Supplement: Supplementary file 4 — Supplementary material 4 (PDF 6 kb) [file 705_2017_3547_MOESM4_ESM.pdf]

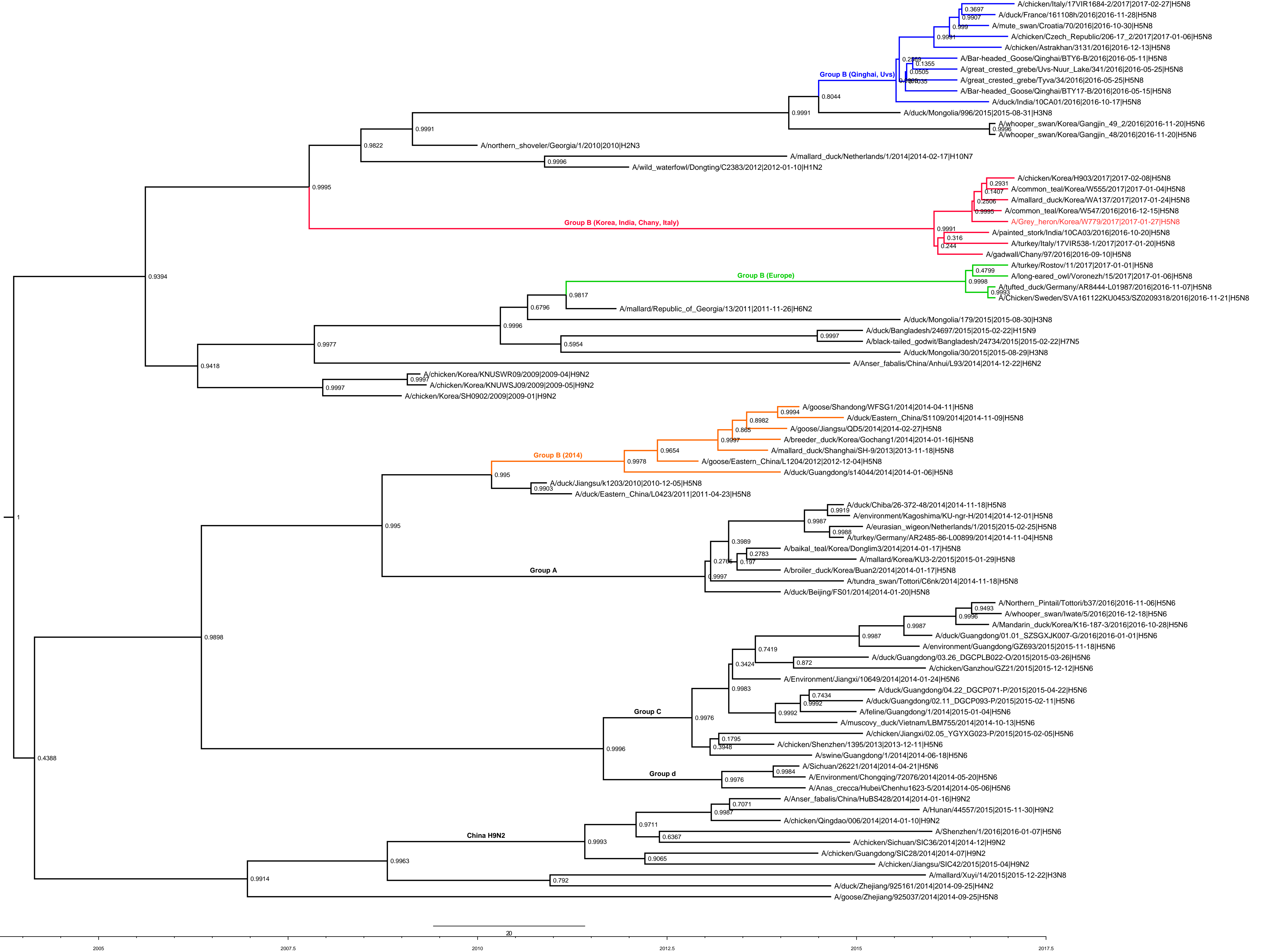

Supplement: Supplementary file 5 — Supplementary material 5 (PDF 8 kb) [file 705_2017_3547_MOESM5_ESM.pdf]

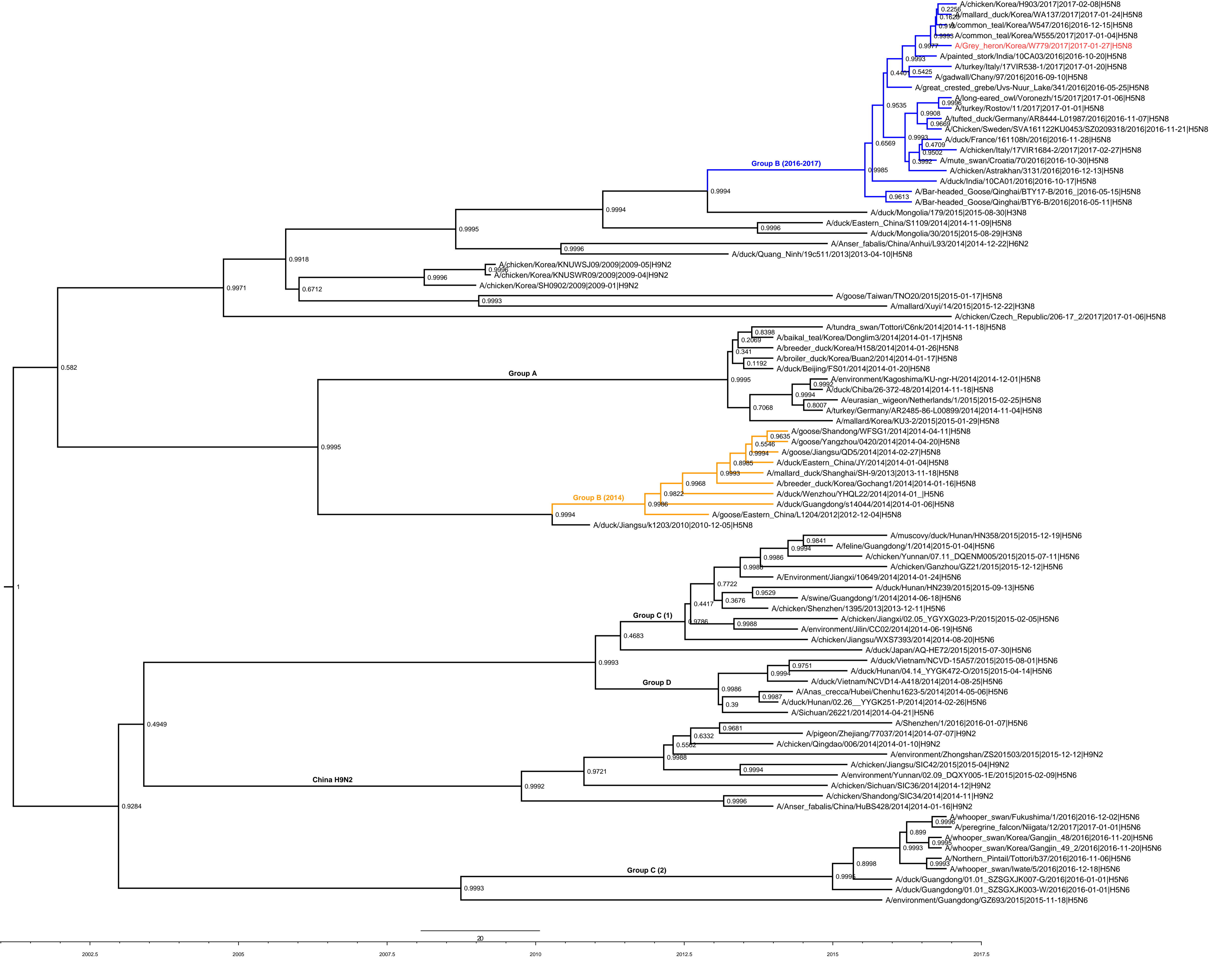

Supplement: Supplementary file 6 — Supplementary material 6 (PDF 8 kb) [file 705_2017_3547_MOESM6_ESM.pdf]

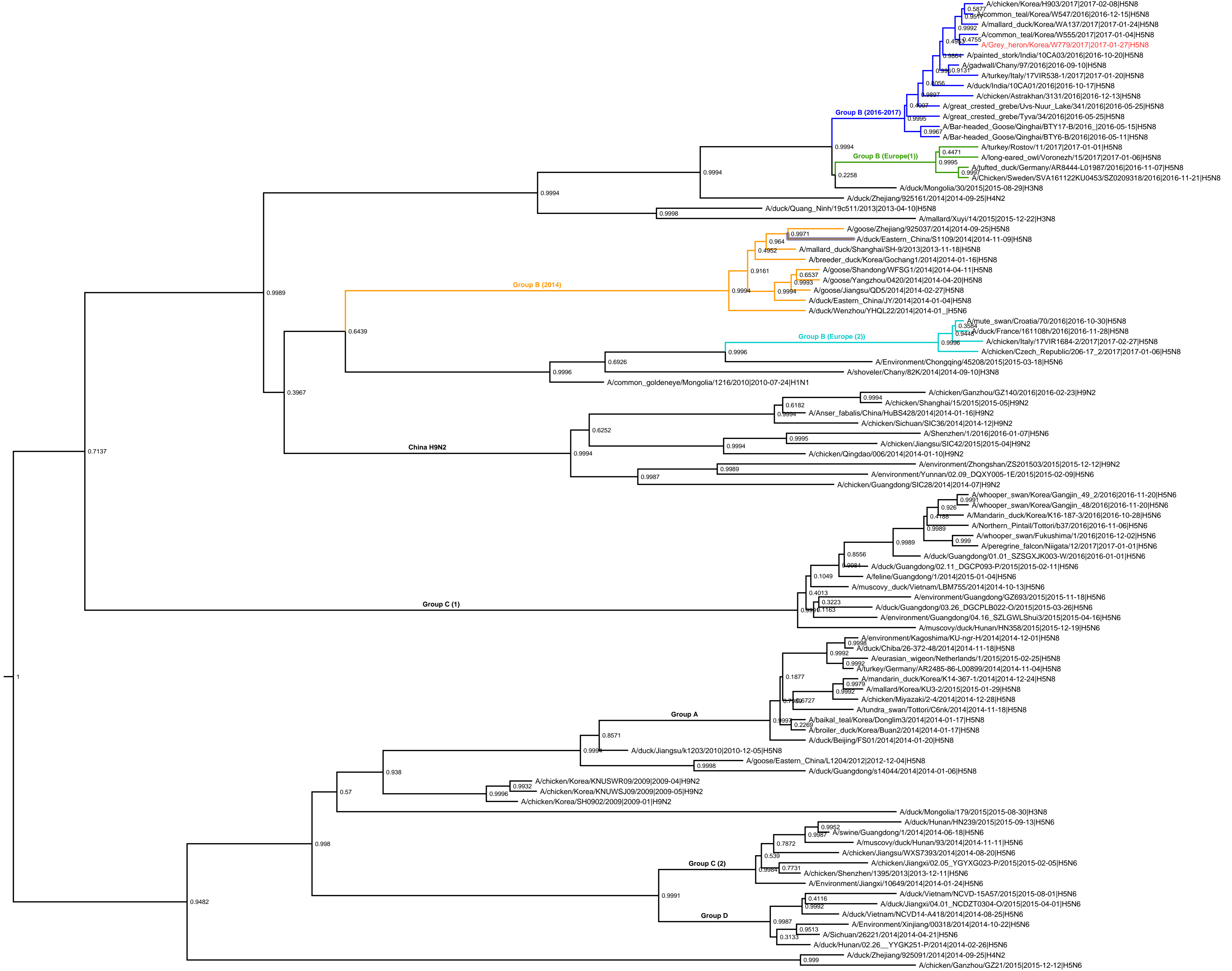

Supplement: Supplementary file 7 — Supplementary material 7 (PDF 9 kb) [file 705_2017_3547_MOESM7_ESM.pdf]
